# Atopic Dermatitis—Beyond the Skin

**DOI:** 10.3390/diagnostics11091553

**Published:** 2021-08-27

**Authors:** Mădălina Mocanu, Dan Vâță, Anisia-Iuliana Alexa, Laura Trandafir, Adriana-Ionela Patrașcu, Mădălina Florina Hâncu, Laura Gheucă-Solovăstru

**Affiliations:** 1 Department of Oral Dermatology, “Grigore T. Popa” University of Medicine and Pharmacy, 700115 Iasi, Romania; 2 Department of Dermatology, “Grigore T. Popa” University of Medicine and Pharmacy, 700115 Iasi, Romania; lsolovastru13@yahoo.com; 3 Department of Ophthalmology, “Grigore T. Popa” University of Medicine and Pharmacy, 700115 Iasi, Romania; 4 Department of Mother and Child Medicine-Pediatrics, “Grigore T. Popa” University of Medicine and Pharmacy, 700115 Iasi, Romania; trandafirlaura@yahoo.com; 5 Dermatology Clinic, “St. Spiridon” County Emergency Clinical Hospital, 700111 Iași, Romania; patrascuai@yahoo.com (A.-I.P.); madalinahancu92@yahoo.com (M.F.H.)

**Keywords:** atopic dermatitis, chronic inflammation, comorbidities, extra-cutaneous manifestations

## Abstract

Atopic dermatitis is a chronic inflammatory disease that can arise during the first months of life or at maturity and have a significant negative impact on the quality of life. The main pathogenic mechanism is the breakdown of cutaneous barrier integrity, which is associated with systemic inflammatory immunologic disorders. Atopic dermatitis involves numerous immunologic, allergic, respiratory, and ophthalmologic comorbidities that develop through similar intricate pathogenic phenomena. The atopic march represents the evolution in time of various allergic diseases, of which food allergies often cause the first manifestations of atopy, even from a very young age. Chronic inflammation translated through specific markers, next to increased immunoglobulin E (IgE) serum levels and heterogenous clinical manifestations, argue for the inclusion of atopic dermatitis in the systemic disease category.

## 1. Introduction

Atopic dermatitis is a common chronic inflammatory disease that mostly afflicts the skin [1]. All aspects of it, from its epidemiology and clinical presentation up to therapeutic management and evolution, fall under the category of heterogeneity [2,3]. In this context, the following question arises: is atopic dermatitis a disease or a term for many diseases having a common phenotype?

To regard atopic dermatitis as a systemic inflammatory disease is still controversial [4], and in this work, we synthetize data from the literature on atopic dermatitis and extra-cutaneous-associated disorders as well as recently discovered pathogenic mechanisms to give a broader picture of atopic dermatitis. As a result, we hope to improve therapeutic methods, discover other innovative therapies, and increase of the life quality of the patients with atopy. The present work is based on searches of international data bases using key words such as “atopy”, “dermatitis”, “inflammation”, “allergy”, and “comorbidities”. The search was limited to human studies written in English. 

## 2. Clinical Cutaneous Manifestations

Atopic dermatitis usually begins within the first 6 months (45% of cases) or during childhood, and up to age 5 (70%) [5]. Outbreaks alternate with periods of greater or lesser remission, and the persistence of specific symptoms until maturity gives atopic dermatitis its chronic nature. Up to 50% of the children diagnosed with it show signs of disease in adulthood. Currently, approximately 20% of children and 10% of adults worldwide suffer from atopic dermatitis, and incidences are increasing in industrialized countries [6,7].

The negative psychosocial and financial impacts are comparable to those associated with epilepsy or diabetes. Beyond the specific symptoms of the disease, insomnia caused by pruritus and a decrease in attention and concentration also contribute to increased absenteeism from work, which leads to economic loss [8,9].

**Figure 1:**
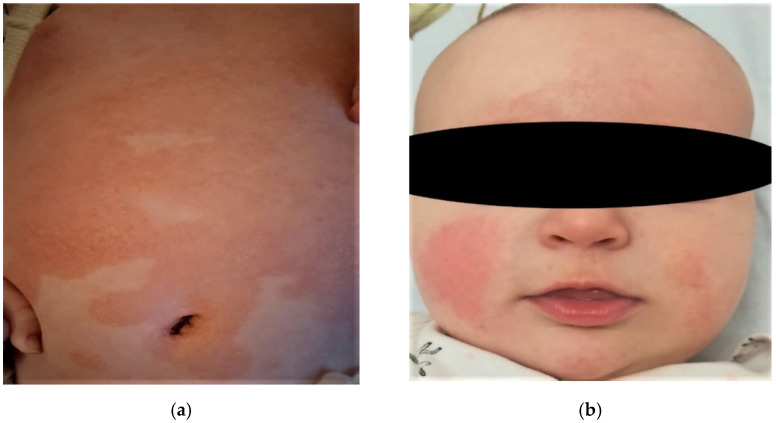
(**a**) Lesions appears to be erythematous plaques with well-defined borders and covered with fine scales and xerotic and itchy areas, extensively involving the trunk and abdomen. (**b**) Symmetric erythematous plaques accompanied by xerosis, pruritus, and fine non-adherent scales involving the face of a baby (cheekbones, chin, forehead).

At the cutaneous level, atopic dermatitis develops through imperfectly delimited erythema accompanied by pruritus, and vesicles in acute stages evolve to chronicity and liquefaction. Xerotic skin is recurrent and often occupies vast areas (Figure 1). The association between xerosis and pruritus leads to the appearance of post-scratch abrasions, which increases susceptibility to infection. Atopic dermatitis can affect any area of the skin, and lesions are distributed symmetrically, especially in flexible regions. The morphology and the location mostly depend on the age of onset, for example: for babies it occurs mainly on the face, neck, scalp, elbows, or knees (Figure 1) [10,11,12].

## 3. Extra-Cutaneous Clinical Manifestations

### 3.1. Allergic Rhinitis

The most explicit evidence that atopic dermatitis is a systemic disease is the so-called “atopic march”, defined as the progress of various allergic diseases over time [13,14]. Atopic dermatitis and food allergies are often the first manifestations of atopy from a very young age. An increasing number of studies show that such children develop allergic rhinitis and allergic asthma; therefore, dermatitis precedes sensitivity to aeroallergens [4]. Up to 50% of the patients with atopic dermatitis will develop asthma, which will later be associated with allergic rhinitis; however, the risk is probably overestimated because the majority of studies have included patients with severe forms of atopy, most of whom required hospitalization [15]. The fundamental pathogenic mechanism of atopy is immune hypersensitivity caused by the rise in IgE serum levels, which promote and sustain food, cutaneous, and respiratory allergic reactions [16]. The classic trio—atopic dermatitis, bronchial asthma, and allergic rhinitis—have a great number of common pathophysiological traits, including abnormal cyclic nucleotide adjustments, mutated immune cells, and mediators of inflammation for similar allergens. Even so, recent studies demonstrated that type 2 atopic dermatitis is more independent of atopy and associated immunologic diseases. The type 2 immune response is characterized by high levels of synthesized interleukins (IL-4, IL-5, IL-9, IL-13, IL-31) and increased serum levels of mastocytes, basophils, and T-helper cells (Th2). For patients with associated respiratory disorders, the identification of type 2 inflammation is important for choosing a target therapy [17,18].

Allergic rhinitis is a pathological condition resulting from the complex interaction of genetic and environmental factors. It is characterized by acute or chronic inflammation of the nasal mucosa brought on by an exaggerated immunologic reaction to aeroallergens in which specific IgE-type antibodies participate. The most well-known chemical mediators of the inflammatory reaction are the histamine and cysteine leukotrienes. A meta-analysis ascertained that of 40.5% of atopic dermatitis patients have rhinitis [19,20]. An increased prevalence of the rhinitis was also found for patients with early-onset atopic dermatitis (<2 years) compared to those in adolescence or adulthood. Recent studies showed that those who develop it later in childhood have milder atopic symptoms because of lower IgE levels. 

A German study emphasized the implication of genetic factors in the association between allergic rhinitis and atopic dermatitis. In both cases, patients exhibited a mutation of the fillagrin gene, which was found to increase the risk of rhinitis regardless of the severity of dermatitis or age of onset [21,22].

The clinical symptoms of allergic rhinitis—sneezing, nasal pruritus, watery rhinorrhea, nasal obstruction, hyposmia/anosmia, and conjunctivitis (tearing, itching, redness of the conjunctiva, and palpebral edemas)—accompany the nasal ones, especially in sensitivity to pollen [23]. The appearance of these symptoms in a patient with atopic dermatitis compels supplementary allergenic investigations. The diagnosis of the allergy is based on the history of the disease and the patient’s medical history and conducted by in vivo or in vitro allergy tests. Historical data comprise the trigger and aggravating factors, responses to the previous treatments, and personal and family history of atopic conditions (e.g., dermatitis, food allergies, rhinitis, and asthma/recurrent wheezing). The most important indoor aeroallergens are dust mites: Dermatophagoides pteronyssinus and Dermatophagoides farinae. House dust is a complex mixture consisting of, aside from the domestic dust mites, human and pet scaly patches, carpet and clothing fibers, insects, molds, sand particles, scraps, and other inert dusts. Other interior allergens come from pets (e.g., dogs, cats, hamsters, Guinea pigs). In Romania, the most important exterior aeroallergen is pollen [24]. The allergenic assessment of rhinitis emphasizes sensitivity to a European-recommended panel of aeroallergens. The skin scratch test is a first-line method for detecting IgE hypersensitivity and can be made using stabilized allergenic extracts. If the scratch test is negative, equivocal, or contra-indicated, the allergen-specific IgE test is an alternative method. There are a multitude of allergen panels, but the one selected must be used in accordance with the patient’s history [25,26,27].

### 3.2. Allergic Bronchial Asthma

The relation between allergic rhinitis and asthma or other inflammatory respiratory conditions is summed up in the concept of “sole disease of the airways”. Statistically, 29–35% of children with atopic dermatitis develop allergic bronchial asthma, and children with atopic dermatitis who contract rhinitis have a significantly higher risk (99.3% of developing allergic bronchial asthma). An important aspect in the pathophysiology of asthma is bronchial hyper-reactivity, which is also present in patients with atopic dermatitis but absent in the criteria for a diagnosis of asthma [28,29].

These studies and their outcomes suggest a powerful similarity between the pathogenesis of allergic bronchial asthma and that of atopic dermatitis. The main etiologic agents for both are aeroallergens, and atopic dermatitis lesions can be aggravated by exposure or direct contact. Immunologic evidence supporting the negative impact of exposure to respiratory allergens is represented by increased serum levels of antigen-specific IgE, which binds to the antigen-presenting cells and to allergen-specific T cells of the damaged skin. Thus, the loss of the epithelial integrity becomes an important mechanism in the pathogenesis of atopic dermatitis and asthma [30,31].

A vast Danish research study demonstrated associations between a mutation on the gene that encodes protocadherin-1 (PCDH1) and asthma, wheezing, and atopic dermatitis in children. An interaction between PCDH-1 and passive exposure to smoke during childhood in the development of asthma was also suggested [29]. Other studies emphasized the correlation among the age of onset for atopic dermatitis, its severity, and the appearance of asthma and allergic rhinitis. At ages <2 years, the onset of atopic dermatitis and moderate-to-severe clinical symptoms imply a higher risk of developing respiratory allergies. These are found mostly in school-aged children, whereas atopic dermatitis appears mostly in early childhood. Increased serum levels of IgE are found in both age groups. Early desensitizing during childhood dramatically reduces these associations [32,33].

Patients with bronchial asthma and atopic dermatitis showed 70% positive results in sensitivity tests for house dust. The clinical picture of bronchial asthma included a series of variable symptoms: respiratory difficulties, thoracic pain, dry cough, wheezing, and sleep disturbances. These symptoms had a series of features with increased specificity for allergic asthma: intensified symptoms at night and in the early morning; sometimes spontaneous remission; aggravation by cold, humidity, physical effort, dust, and pollen; sudden onset of acute episodes; or seasonal evolution. The stages of the diagnostic algorithm of bronchial asthma consist of a detailed analysis of the personal and family medical history (antecedents of atopy, atopic dermatitis, allergic rhinitis, food allergies), a complete physical exam including a pneumological consult and cutaneous testing for specific allergens, and the spirometric measurement of the respiratory volume [34,35].

### 3.3. Food Allergies

During the last decades, remarkable progress has been made regarding pathogenic mechanisms, diagnostic criteria, and the treatment protocol for patients with atopic dermatitis, of whom 20–40%, on average, have food allergies [36]. These manifest clinically in various ways depending on age, severity of the associated atopic comorbidities, and responses to medication. The relation between atopic dermatitis and food allergy is based on a series of pathogenic mechanisms, most of them being IgE-mediated mechanisms or through type 2 inflammatory immunologic abnormalities. In the literature, two theories refer to this association: in the first, the food allergy may cause atopic dermatitis or be a factor that worsens an atopy; in the second, atopic dermatitis induces the food allergy. Most studies show that food allergens have a role at the start of atopic dermatitis or worsen existing symptoms, thus giving the appearance of a severe form of the disease [37,38,39].

The gold standard for diagnosing food allergy, regardless of its origin, is the provocation test. On the other hand, for example, a newborn with severe atopic dermatitis develops multiple food sensitivities that can be emphasized either through the presence of big titers of specific IgE in the serum or through positive skin scratch tests. The main food allergens implicated in atopic dermatitis in babies are eggs, dairy products, and nuts. Eggs, and in a smaller amount, dairy products, are responsible for the aggravation of eczemas; nuts are especially involved in immediate, and even anaphylactic allergic reactions [40].

The onset of a food allergy can take a few minutes or up to 2–4 h after ingestion mediated by IgE. Rarely, late eczema reactions appear, but these are not mediated by IgE and their pathogenesis is unclear. They develop after 2–6 days and can appear as an isolated phenomenon or as part of the immediate reaction. At the cutaneous level, they present as papules accompanied by pruritus and rash, contact dermatitis or, in the severe cases, as an angioedema. Aside from dermatological manifestations, food allergies can induce oropharyngeal, gastro-digestive, respiratory, and cardiovascular disorders. The heterogeneity of the clinical forms of a food allergy involves a provocation for establishing the diagnosis and adequate management. The beneficial effects of the elimination diet on atopic dermatitis lesions have been demonstrated, but a monitored nutritional program is recommended, especially for children. Avoiding the appearance of nutritional deficiencies is intentional, so the reintroduction of restricted foods must be made gradually to avoid the risk of an anaphylactic reaction. The frequency of food allergies decreases with the age [41,42].

### 3.4. Ophthalmic Pathology

Although studies referring to the association between atopic dermatitis and ophthalmic diseases are relatively limited, some scientific evidence attests to modifications in the ocular surface in atopic patients. Of these, the most common are the blepharitis, conjunctivitis, uveitis, keratoconus, and rarely, detached retinas. Approximately 25–50% of patients with atopic dermatitis develop ocular complications over time [43].

Ocular allergies present a heterogenous group of manifestations that predominantly affect the conjunctiva and are determined by an exaggerated immune response to certain allergens. More mechanisms to explain the association between atopy and the conjunctivitis were proposed. Thus, the interaction between the mechanical stress induced by pruritus, the loss of the integrity of the cutaneous barrier, and modifications to the local microbiome contribute to the development of ocular complications or worsening of the pre-existing ones. Seasonal and perennial conjunctivitis are characterized by inflammation due to type 1 hypersensitivity (IgE mediated), while inflammation from vernal and keratoconjunctivitis are mediated by Th2 helper lymphocytes. Eosinophilic cellular infiltration and tissue remodeling in keratoconjunctivitis may produce severe corneal lesions that can lead to blindness [44,45].

Ocular pruritus, frequent in atopic patients, is the product of germinal colonization by local trauma and reduced antimicrobial cutaneous protection. Incidences of bacterial colonization of the conjunctival sac and eyelid margins is significantly greater compared to people without atopy (86% vs. 25%). The main etiologic agent of ocular infections in atopic dermatitis patients is *Staphylococcus aureus* (67% of cases) [46,47].

Pruritus ophthalmic conditions, like conjunctivitis or blepharitis, can be complicated by keratoconus, the non-inflammatory progressive thinning of the cornea characterized by astigmatism, bilateral ectasy, and, in the final stage, scarring. The rate of incidence varies between 0.5% and 39%, depending on the severity of atopic lesions, especially on the face and eyelids. A 1977 study emphasized that approximately 35% of patients with keratoconus have an atopic tendency. Chronic inflammation in both atopic dermatitis and keratoconus is also associated with mutations on the fillagrin gene [48,49].

The ocular pruritus is the main trigger involved in the etiopathogenesis of retinal detachment. Lesions induced by the pruritus on the periphery of the retina may be considered similar to traumatic ones [50]. IgE serum levels do not have a direct impact on the retina detachment, but pigmentation of the angle of the anterior room of the eye is considered a risk indicator in patients with atopic dermatitis, which is the reason that periodic examination of the eye fundus is recommended [51].

Cataracts are usually bilateral, symmetrical, and appear in the posterior and anterior subcapsular regions. The progression of the disease depends on certain factors, such as ocular pruritus and severity of facial skin lesions. An association between the development of cataracts and increased IgE serum levels, the duration of the topic cortical therapy at the facial level, and systemic corticoid treatment was not demonstrated. A Danish studio established a correlation between atopic dermatitis and cataracts that especially applies to patients under 50 [52,53].

### 3.5. Digestive Disorders

Gastroenterological impairment one of the extra-cutaneous pathologic manifestations associated with atopic dermatitis. It is especially common in children and, in most cases, associated with elevated serum IgE levels. Moreover, increased levels of reactive immunoglobulins were demonstrated in duodenal juice samples taken from patients with atopy. Specific IgE stimulation was promoted by the increased transfer of antigens to the functionally deficient intestinal mucosa. This mechanism demonstrated both the possibility of an association between digestive disorders and atopy, and the possible involvement of digestive tract abnormalities in the etiopathogenesis of atopic dermatitis. In current practice, gastrointestinal symptoms often precede clinical skin signs in children with atopic eczema. The disruption of the integrity of the intestinal membrane in the context of immunological hypersensitivity is based on the production and maintenance of local inflammation. Therapies aimed at decreasing membrane permeability have also proven effective in improving the severity of skin lesions in atopic dermatitis. These results confirm the association between digestive morpho-functional disorders and atopy [54].

Eosinophilic gastroenteritis is a relatively rare digestive disorder characterized by eosinophilic infiltration of the stomach and small intestine and appears mainly in atopic patients or in those with pre-existing autoimmune diseases. The clinical picture is characterized by abdominal pain, nausea, vomiting, and diarrhea. Biologically, the predominant symptom is anemic syndrome induced by malabsorption, enteropathy, and protein deficit. The high frequency of atopy in patients with intestinal inflammatory diseases is based on the type 2 inflammatory mechanism [55].

Eosinophilic esophagitis is a chronic inflammatory disease caused by a specific immune response to a particular allergen that leads to progressive esophageal dysfunction. Epidemiological associations between eosinophilic esophagitis and other allergic manifestations are well known and probably induced by the interaction between a number of common genetic, environmental, and immunological factors. Atopic dermatitis, IgE-mediated food allergy, asthma, and allergic rhinitis confer a cumulative individual risk for the development over time of allergic eosinophilic esophagitis. In view of these issues, it is currently proposed as the fifth member of the atopic march [56].

Inflammatory bowel diseases are a group of chronic inflammatory diseases based on appropriate immune responses that clinically affect the gut and occur in individuals with genetic susceptibility. The association between these and atopic dermatitis is explained by a number of common pathogenic features: immune dysfunction that generates a chronic pro-inflammatory status, common genetic mutations, and microbiota disorder. Immunologically, both pathological entities involve Th2 cell-related response abnormalities. Genetically, a common gene increases susceptibility to epithelial inflammation in the skin and in the epithelium of the intestinal mucosa. We refer to the single nucleotide risk allele A (SNP) rs7927894 located on chromosome 11q13.5. Alternation to the intestinal microbiome and the low diversity of intestinal microbiota not only contribute to the development of allergic phenomena and food intolerance, but also to cutaneous manifestations of atopic dermatitis [57].

### 3.6. Autoimmune Diseases

Increased incidence of autoimmune diseases in patients with atopy is well known; moreover, recent studies consider atopic dermatitis as an important risk factor for autoimmune development. The prevalence of the immune disorders, especially of the thyroid, is higher in children with atopic dermatitis, especially those with lactose intolerance. A review of the epidemiological data regarding rheumatoid arthritis, multiple sclerosis, and type I diabetes mellitus suggests that type Th1-mediated inflammation can have a protective effect against atopy, and that atopy could reduce its severity, but not necessarily the appearance of autoimmune diseases [58].

Alopecia areata is a non-scarring form of hair loss based on autoimmune mechanisms directed against hair follicles. The inflammatory reaction is mainly type 1, but is also associated with the production of Th2-type cytokines similar to those in atopic dermatitis. Other atopic comorbidities of alopecia areata based mainly on type 2 inflammation are rhinitis, allergic asthma, and eczema. Seasonal exacerbations of alopecia areata were observed, especially in autumn, with a pattern similar to the seasonal evolution in the severity of atopic dermatitis.

Vitiligo is an autoimmune disorder characterized by the destruction of melanocytes and the consequent loss of pigmentation. The association between vitiligo and other immune diseases has been intensively studied, and it seems that the common pathogenic element is chronic inflammation. Meta-analyses found that patients with a history of atopic dermatitis have a 2.5 times higher risk of developing alopecia areata and 7.5 times the risk of vitiligo compared to non-atopic individuals. Therefore, atopic dermatitis becomes a risk factor for the development of alopecia areata or vitiligo over time [59,60].

Juvenile idiopathic arthritis (JIA) is another autoimmune and inflammatory disease with an increased risk of developing in allergic children. The complex link between both allergic and autoimmune diseases involves an interaction between Th1-mediated JIA and Th2-mediated allergic diseases. In 2016, Lin et al. published the first study on children in which they showed that childhood-onset allergic diseases are a risk factor for JIA [61]

Atopic dermatitis and psoriasis vulgaris are chronic inflammatory skin diseases, with different clinical manifestations caused by a series of immunological dysfunctions with a major negative psychosocial and financial impact. The presence of both these pathologies in the same patient is rare, and there is still little information about the clinical evolution of patients with both diseases. Their coexistence in the same person is due to one of two scenarios: (1) the alternation of periods of activity and remission: one starts, the other disappears, and vice versa; (2) the appearance at different stages of life, for example: atopic dermatitis may occur in childhood, then go into remission, to be followed by psoriasis in adulthood. The second hypothesis is the more common one [62].

From a pathogenic point of view, the mechanisms of these two diseases are opposite, which limits the possibility of their association and the analysis that explains their coexistence in certain patients. Atopic dermatitis occurs through an immunological mechanism mediated by Th2 cells and increased serum levels of total and specific IgE; psoriasis is caused by an aberrant immune response driven by Th17 cells that secrete high serum levels of interleukin-17A, interleukin-17F, and interleukin-22. These observations suggest that a distinct subset of antigen-specific T cells determines the pathogenesis of psoriasis and eczema. In addition to the barrier function of the skin being affected in both conditions, the systemic immune response mediated by T lymphocytes involves damage to the skin and other sites, such as the osteo-articular system (psoriatic arthropathy) or airways (asthma, rhinitis), which are observations that support the idea that each pathological condition is a systemic disease [63].

The relation between autoimmunity and atopy is generally based on an exaggerated reaction in patients with high IgE levels more than on a systemic inflammatory reaction. The first evidence of IgE autoantibodies directed against human proteins was described 25 years ago, when they were detected in the serum of patients with atopic dermatitis or were determined by a positive response to autoantigens in vitro. However, the term “autoimmunity” has never been claimed, as dermatitis is primarily an atopic disease. This IgE-mediated self-activity has been termed “autoallergy”, and its impact on the severity of the disease is still the subject of extensive research [64].

Patients with high serum levels of total or specific IgE for environmental allergens tend to develop severe forms of atopy, and the impact of the immune response against autoproteins is difficult to estimate. Extensive studies involving 2644 subjects diagnosed with atopic dermatitis revealed the presence of an IgE-type reaction in 23–91% of cases in a wide panel of food and respiratory and microbial allergens, but it was difficult to estimate the proportion of IgE antibodies directed against “self” proteins.

In a study of 346 children with atopic dermatitis and 117 controls with elevated serum levels of antinuclear antibodies (ANA), both groups showed a tendency to increase ANA antibodies over time, but children with dermatitis ANA antibodies reached high values more quickly. These findings argue for the idea that patients with early-onset atopy in childhood have a higher and earlier risk of developing an autoimmune disease.

Understanding the contribution of IgE autoantibodies in the pathophysiology of atopic dermatitis, knowing the targets, and studying the correlation between auto-IgE and the severity of the disease will lead to the prompt diagnosis and individualized management of atopic dermatitis and possible autoimmune comorbidities [65,66].

### 3.7. Psychological Comorbidities

Atopic dermatitis is regarded as a chronic pathology with fluctuating outbreaks and remissions, and has a negative psychological impact on children and adults diagnosed with this disease. In 90% of cases, it begins before 5 years of age and affects about 1 in 5 children. They feel the burden of physical suffering, but also psychological changes such as mood swings, sleep and behavioral disorders, attention deficit, anxiety, depression, and social isolation, especially in adolescence. Their families also face the financial burden of treatment and the difficulty of managing the consequences of sleep deprivation and the socio-negative visual impact of the disease [4].

Psychological factors are considered provocative, and their control is part of the general management of the disease, both in adults and children. In recent decades, a large number of studies showed that patients with atopic dermatitis typically have a lower quality of life, anger problems, hostility, and poor marital relationships.

Atopic dermatitis is considered to be a disease with a psychosomatic component in which stress is a key factor in triggering and maintaining its outbreaks. Stress induces an increase in the permeability of the skin barrier and has the potential to stimulate systemic inflammation by releasing proinflammatory neuropeptides. On the other hand, neuropeptides released in the skin of atopic dermatitis patients influence the central nervous system, disrupting recognition and perception functions, as well as behavior. The negative visual impact of the disease is itself a stress factor with a strong negative psychosocial influence. Research has shown that the central and peripheral nervous system in association with the immune system play an important role in modulating the expression of various dermatological diseases. Thus, a new concept of the neuro-immuno-cutaneous system was developed [67,68].

## 4. Oral Manifestations Associated with Atopic Dermatitis

Many cutaneous or systemic pathologies based on immune mechanisms (lichen planus, lupus erythematosus, inflammatory bowel disease, psoriasis vulgaris, bullous dermatosis, atopy) have clinical manifestations in the oral mucosa. Often, their onset is in the oral cavity [69].

The correlation between oral lesions and atopy is not new, but the pathogenic mechanisms underlying this association have not been fully elucidated, and studies addressing this topic are few. In addition to skin, respiratory, or digestive impairment, atopic patients may experience changes in the oral mucosa. For those who have personal or family medical history of atopy, studies have reported an increased susceptibility to allergies, elevated serum IgE levels, and the development of benign migratory glossitis (geographical tongue). It seems that chronic inflammation is the common process that underlies this association. Benign migratory glossitis is a recurrent disease characterized by alternating episodes of exacerbation and remission. It is clinically characterized by linear hyperkeratosis delimiting an erythematous region with hypotrophic filiform papillae, mainly on the dorsal face and edges of the tongue. It is usually asymptomatic, but some patients report pain or burning sensation, especially after ingesting acidic or spicy food [70].

Although increased frequency of atopy (dermatitis, asthma, rhinitis) in patients with geographic tongue has been described by Marks et al., benign migratory glossitis is not a specific sign of atopy and can be found it in other conditions such as psoriasis, vitamin deficiency, and liver disease [71].

Atopic cheilitis (inflammation of the lips) is a minor diagnostic criterion for atopy. It has the clinical appearance of erythema (reddening) of the lips, accompanied by scales, accentuated radial folds, and irritative commissural lesions (angular cheilitis). Most cases of atopic (allergic) cheilitis are due to a contact sensitivity frequently induced by cosmetics such as lipsticks or lip balms [72]. However, the possibility of “sicca” cheilitis in association with atopic dermatitis should also be considered.

Other oral manifestations of atopy are the absence, or the low number of, Fordyce granules, which are normally present on the labial and jugal mucosa in healthy subjects, irritative stomatitis, recurrent aphthous stomatitis, fungiform papillary hypertrophy, and oral candidiasis [73]. None of these pathological conditions is specific to atopy, but the correlation with atopic status has been proven by decades of studies.

## 5. Conclusions

Atopic dermatitis may be a condition of the skin, but scientific evidence over the past decades supports the concept that it is also a systemic disease. The impairment of the cutaneous barrier, chronic inflammation, and IgE-mediated hypersensitivity are the main mechanisms that contribute to the appearance of a pathological condition that affects multiple organs and systems. Research into the intimate mechanisms between atopy and various extra-cutaneous disorders are in a continuous development and still represent a challenge for the specialists. This paper highlighted the tendency of atopic patients to associate cutaneous, respiratory, renal, autoimmune, and ophthalmological manifestations in a heterogeneous clinical picture that is difficult to diagnose and manage. The clinical aspects are complex considering the possibility of the onset from the first months of life and the fluctuating progression of the disease with alternating periods of exacerbations and remission. It is important to know all aspects of this disease to develop a diagnostic and treatment guide for specialists in the management of atopy. The global, multidisciplinary approach of atopic dermatitis seen as a systemic disease, not only a dermatologic one, has tremendous importance for prevention, diagnosis, and targeted therapeutic strategies.

## References

[1] Oliveira C., Torres T. (2019). More than skin deep: The systemic nature of atopic dermatitis. Eur. J. Dermatol..

[2] Strom M.A., Silverberg J.I. (2017). Association between atopic dermatitis and extracutaneous infections in US adults. Br. J. Dermatol..

[3] Torres T., Ferreira E.O., Goncalo M., Mendes-Bastos P., Selores M., Filipe P. (2019). Update on Atopic Dermatitis. Acta Med. Port..

[4] Darlenski R., Kazandjieva J., Hristakieva E., Fluhr J.W. (2014). Atopic dermatitis as a systemic disease. Clin. Dermatol..

[5] Serrano L., Patel K.R., Silverberg J.I. (2019). Association between atopic dermatitis and extracutaneous bacterial and mycobacterial infections: A systematic review and meta-analysis. J. Am. Acad. Dermatol..

[6] Carlsten C., Dimich-Ward H., Ferguson A., Watson W., Rousseau R., Dybuncio A., Becker A., Chan-Yeung M. (2013). Atopic dermatitis in a high-risk cohort: Natural history, associated allergic outcomes, and risk factors. Ann. Allergy Asthma Immunol..

[7] Klasa B., Cichocka-Jarosz E. (2020). Atopic Dermatitis—Current State of Research on Biological Treatment. J. Mother Child.

[8] Plotz S.G., Ring J. (2010). What’s new in atopic eczema?. Expert Opin. Emerg. Drugs.

[9] Silverberg J.I. (2018). Associations between atopic dermatitis and other disorders. F1000 Res..

[10] Thestrup-Pedersen K. (2000). Clinical aspects of atopic dermatitis. Clin. Exp. Dermatol..

[11] Wang X., Shi X.D., Li L.F., Zhou P., Shen Y., Song X.K. (2017). Prevalence and clinical features of adult atopic dermatitis in tertiary hospitals of China. Medicine.

[12] Kim J., Kim B.E., Leung D.Y.M. (2019). Pathophysiology of atopic dermatitis, Clinical implications. Allergy Asthma Proc..

[13] Hogan M.B., Peele K., Wilson N.W. (2012). Skin barrier function and its importance at the start of the atopic march. J. Allergy.

[14] Schneider L., Hanifin J., Boguniewicz M., Eichenfield L.F., Spergel J.M., Dakovic R., Paller A.S. (2016). Study of the atopic march: Development of atopic comorbidities. Pediatr. Dermatol..

[15] Selene K., Bantz Z.Z., Tao Z. (2014). The Atopic March: Progression from Atopic Dermatitis to Allergic Rhinitis and Asthma. J. Clin. Cell Immunol..

[16] Wade W., Sandeep K. (2011). Atopic dermatitis. Allergy Asthma Clin. Immunol..

[17] Weidinger S., Beck L.A., Bieber T., Kabashima K., Irvine A.D. (2018). Atopic dermatitis. Nat. Rev. Dis. Primers.

[18] Lee H.H., Patel K.R., Singam V., Rastogi S., Silverberg J.I. (2019). A systematic review and meta-analysis of the prevalence and phenotype of adult-onset atopic dermatitis. J. Am. Acad. Dermatol..

[19] González-Mendoza T., Bedolla-Barajas M., Bedolla-Pulido T.R., Morales-Romero J., Pulido-Guillén N.A., Lerma-Partida S., Meza-López C. (2019). The prevalence of allergic rhinitis and atopic dermatitis in late adolescents differs according to their gender. Rev. Alerg. Mex..

[20] Izquierdo-Dominguez A., Jauregui I., Del Cuvillo A., Montoro J., Davila I., Sastre J., Bartra J., Ferrer M., Alobid I., Mullol J. (2017). Allergy rhinitis: Similarities and differences between children and adults. Rhinology.

[21] Jonathan S., Joel M.G., David J.M., Mark B., Luz F., Mitchell H.G., Eric L.S., Peck Y.O., Zelma C.C. (2018). Association of atopic dermatitis with allergic, autoimmune, and cardiovascular comorbidities in US adults. Ann. Allergy Asthma Immunol..

[22] Knudgaard M.H., Andreasen T.H., Ravnborg N., Bieber T., Silverberg J.I., Egeberg A., Halling A.-S., Thyssen J.P. (2021). Rhinitis prevalence and association with atopic dermatitis: A systematic review and meta-analysis. Ann. Allergy Asthma Immunol..

[23] Langdon C., Guilemany J.M., Valls M., Alobid I., Bartra J., Dávila I., Del Cuvillo A., Ferrer M., Jáuregui I., Montoro J. (2016). Allergic rhinitis causes loss of smell in children: The OLFAPEDRIAL study. Pediatr. Allergy Immunol..

[24] Scadding G.K., Kariyawasam H.H., Scadding G., Mirakian R., Buckley R.J., Dixon T., Durham S.R., Farooque S., Jones N., Leech S. (2017). BSACI guideline for the diagnosis and management of allergic and non-allergic rhinitis. Clin. Exp. Allergy.

[25] Armengot-Carbo M., Hernández-Martín Á., Torrelo A. (2015). The role of filaggrin in the skin barrier and disease development. Actas Dermosifiliogr..

[26] Incorvaia C., Cavaliere C., Frat F., Masieri S. (2018). Allergic rhinitis. J. Biol. Regul. Homeost. Agents.

[27] Dierick B.J.H., Flokstra-de Blok B.M.J., Muraro A., Postma M.J., Kocks J.W.H., van Boven J.F.M. (2020). Burden and socioeconomics of asthma, allergic rhinitis, atopic dermatitis and food allergy. Expert Rev. Pharmacoecon. Outcomes Res..

[28] Mastrorilli C., Posa D., Cipriani F., Caffarelli C. (2016). Asthma and allergic rhinitis in childhood: What’s new. Pediatr. Allergy Immunol..

[29] Zaripova T.N., Antipova I.I., Reshetova G.G. (2019). Features of the current of bronchial asthma in the presence of komorbidy allergic rhinitis. Ter. Arkh..

[30] Brinkman L., Raaijmakers J.A.M., Bruijnzeel-Koomen K.A.F.M., Koenderman L., Lammers W.J. (1997). Bronchial and skin reactivity in asthmatic patients with and without atopic dermatitis. Eur. Respir. J..

[31] Amat F., Soria A., Tallon P., Bourgoin-Heck M., Lambert N., Deschildre A., Just J. (2018). New insights into the phenotypes of atopic dermatitis linked with allergies and asthma in children: An overview. Clin. Exp. Allergy.

[32] Yang L., Fu J., Zhou Y. (2020). Research Progress in Atopic March. Front. Immunol..

[33] Traidl S., Werfel T. (2019). Atopic dermatitis and general medical comorbidities. Internist.

[34] Reddy A.P., Gupta M.R. (2014). Management of asthma: The current US and European guidelines. Adv. Exp. Med. Biol..

[35] Moral L., Vizmanos G., Torres-Borrego J., Praena-Crespo M., Tortajada-Girbés M., Pellegrini F.J., Asensio Ó. (2019). Asthma diagnosis in infants and preschool children: A systematic review of clinical guidelines. Allergol. Immunopathol..

[36] Marcel M.B., Jean-Christoph C., Mark B., Philippe A.E. (2013). Evaluation of Food Allergy in Patients with Atopic Dermatitis. J. Allergy. Clin. Immunol..

[37] Roduit C., Frei R., Loss G., Buchele G., Weber J., Depner M., Loeliger S., Dalphin M.L., Roponen M., Hyvärinen A. (2012). Development of atopic dermatitis according to age of onset and association with early-life exposures. J. Allergy Clin. Immunol..

[38] Mastrorilli C., Caffarelli C., Hoffmann-Sommergruber K. (2017). Food allergy and atopic dermatitis: Prediction, progression, and prevention. Pediatr. Allergy Immunol..

[39] Sugita K., Akdis C.A. (2020). Recent developments and advances in atopic dermatitis and food allergy. Allergol. Int..

[40] Robison R.G., Singh A.M. (2019). Controversies in Allergy: Food Testing and Dietary Avoidance in Atopic Dermatitis. J. Allergy Clin. Immunol. Pract..

[41] Domínguez O., Plaza A.M., Alvaro M. (2020). Relationship between Atopic Dermatitis and Food Allergy. Curr. Pediatr. Rev..

[42] Brough H.A., Nadeau K.C., Sindher S.B., Alkotob S.S., Chan S., Bahnson H.T., Leung D.Y.M., Lack G. (2020). Epicutaneous sensitization in the development of food allergy: What is the evidence and how can this be prevented?. Allergy.

[43] Marta P., Anna Z.K., Patrycja D., Monika R., Iwona G.L., Marek K. (2020). Ophthalmic manifestations of atopic dermatitis. Adv. Dermatol. Allergol..

[44] Nutten S. (2015). Atopic dermatitis: Global epidemiology and risk factors. Ann. Nutr. Metab..

[45] Sacchetti M., Abicca I., Bruscolini A., Cavaliere C., Nebbioso M., Lambiase A. (2018). Allergic conjunctivitis: Current concepts on pathogenesis and management. J. Biol. Regul. Homeost. Agents.

[46] Guglielmetti S., Dart J.K., Calder V. (2010). Atopic keratoconjunctivitis and atopic dermatitis. Curr. Opin. Allergy Clin. Immunol..

[47] Chisholm S.A.M., Couch S.M., Custer P.L. (2017). Etiology and Management of Allergic Eyelid Dermatitis. Ophthalmic Plast Reconstr. Surg..

[48] Lapp T., Auw-Haedrich C., Reinhard T., Evans R., Rodríguez E., Weidinger S., Jakob T. (2014). Analysis of filaggrin mutations and expression in corneal specimens from patients with or without atopic dermatitis. Int. Arch. Allergy Immunol..

[49] Hsu J.I., Pflugfelder S.C., Kim S.J. (2019). Ocular complications of atopic dermatitis. Cutis.

[50] Yamamoto K., Wakabayashi Y., Kawakami S., Numata T., Ito T., Okubo Y., Tsuboi R., Goto H. (2019). Recent trends of ocular complications in patients with atopic dermatitis. Jpn. J. Ophthalmol..

[51] Kothari N., Young R.C., Read S.P., Tutiven J., Perez V.L., Flynn H.W., Berrocal A.M. (2017). Retinal Detachment Associated with Atopic Dermatitis. Ophthalmic Surg. Lasers Imaging Retina.

[52] Jeon H.S., Choi M., Byun S.J., Hyon J.Y., Park K.H., Park S.J. (2018). Association of Pediatric Atopic Dermatitis and Cataract Development and Surgery. JAMA Ophthalmol..

[53] Ruiz-Lozano R.E., Hernandez-Camarena J.C., Roman-Zamudio M., Alcazar-Félix R.J., Davila-Cavazos O., Cardenas-de la Garza J.A. (2021). Three types of cataract associated with atopic dermatitis and chronic topical corticosteroid use: A case report. Dermatol. Ther..

[54] Vibeke R., Eva B., Niels H.V., Anders P., Kim F.M. (2004). Effect of probiotics on gastrointestinal symptoms and small intestinal permeability in children with atopic dermatitis. J. Pediatr..

[55] Tzanakis N.E., Tsiligianni I.G., Siafakas N.M. (2010). Pulmonary involvement and allergic disorders in inflammatory bowel disease. World J. Gastroenterol..

[56] Peter C., David A.H. (2019). Allergic Comorbidity in Eosinophilic Esophagitis: Mechanistic Relevance and Clinical Implications. Clin. Rev. Allergy Immunol..

[57] Wang C.-H., Fu Y., Chi C.-C. (2020). Association of atopic dermatitis with inflammatory bowel disease: A systematic review and meta-analysis. Dermatol. Sin..

[58] Pedulla M., Miraglia Del Giudice M., Fierro V., Ruocco E. (2012). Atopy as a risk factor for thyroid autoimmunity in children. J. Biol. Regul. Homeost Agents.

[59] Aaron M.D., Jordan M.T., Wen-Qing L., Eunyoung C., Tricia L., Emma G.Y., Abrar A.Q. (2017). Incident alopecia areata and vitiligo in adult women with atopic dermatitis: Nurses’ Health Study 2. Allergy.

[60] Jonathan I.S., Nanette B.S. (2013). Association Between Vitiligo and Atopic Disorders: A Pilot Study. JAMA Dermatol..

[61] Lin C.H., Lin C.L., Shen T.C., Wei C.C. (2016). Epidemiology and risk of juvenile idiopathic arthritis among children with allergic diseases: A nationwide population-based study. Pediatr. Rheumatol. Online J..

[62] Bozek A., Zajac M., Krupka M. (2020). Atopic Dermatitis and Psoriasis as Overlapping Syndromes. Mediat. Inflamm..

[63] Eyerich S., Onken A.T., Weidinger S., Franke A., Nasorri F., Pennino D., Grosber M., Pfab F., Schmidt-Weber C.B., Mempel M. (2011). Mutual Antagonism of T Cells Causing Psoriasis and Atopic Eczema. N. Engl. J. Med..

[64] Roesner L.M., Werfel T. (2019). Autoimmunity (or Not) in Atopic Dermatitis. Front. Immunol..

[65] Badloe F.M.S., Shauni D.V., Coolens K., Schmidt-Weber C.B., Gutermuth R.J., Krohn I.K. (2020). IgE autoantibodies and autoreactive T cells and their role in children and adults with atopic dermatitis. Clin. Transl. Allergy.

[66] Ress K., Metsküla K., Annus T., Putnik U., Lepik K., Luts K., Uibo O., Uibo R. (2015). Antinuclear antibodies in atopic dermatitis: A cross-sectional study on 346 children. Int. J. Dermatol..

[67] Benea V., Muresian D., Manolache L., Robu E., Diaconu J.D. (2001). Stress and Atopic Dermatitis. Dermatol. Psychosom..

[68] Gieler U., Gieler T., Peters E.M.J., Linder D. (2020). Skin and Psychosomatics–Psychodermatology today. J. Ger. Soc. Dermatol..

[69] Gomez-Casado C., Sanchez-Solares J., Izquierdo E., Díaz-Perales A., Barber D., Escribese M.M. (2021). Oral Mucosa as a Potential Site for Diagnosis and Treatment of Allergic and Autoimmune Diseases. Foods.

[70] Dick T.N.A., Teixeira-Souza T., Carneiro S., Moore D., de Ferreira D.C., Pestana S., Boechat J.L., Milagres A., Picciani B. (2018). Geographic tongue and atopy:Is there an association?. Rev. Bras. Odontol..

[71] Marks R., Simons M.J. (1979). Geographic tongue—A manifestation of atopy. Br. J. Dermatol..

[72] Țăranu T., Țăranu T., Toader M.P. (2014). Precis de Dermatologie Generale et Buccale.

[73] Veller-Fornasa C., Bezze G., Rosin S., Lazzaro M., Tarantello M., Cipriani R. (2003). Recurrent Aphthous Stomatitis and Atopy. Acta Derm. Venereol..

